# A note on two novel easy-to-interpret feature effect measures for partial dependence plots in a classification setting

**DOI:** 10.1080/02664763.2025.2554823

**Published:** 2025-09-07

**Authors:** Andreas Karlsson Rosenblad

**Affiliations:** a Regional Cancer Centre Stockholm-Gotland, Region Stockholm, Stockholm, Sweden; b Department of Statistics, Uppsala University, Uppsala, Sweden

**Keywords:** Black box model, effect measure, interpretable machine learning, odds ratio, relative risk, supervised learning models, 62R07, 62G05, 62P10

## Abstract

Classification of observations into one of several distinct categories is a common task in applied statistics, traditionally performed using parametric statistical models such as logistic regression. These parametric models are, however, often outperformed in terms of prediction accuracy by black box supervised learning models (BBSLMs). A drawback of BBSLMs is the lack of easy-to-interpret feature effect measures similar to the odds ratio (OR) for logistic regression models. The present paper derives two novel feature effect measures based on partial dependence plots for binary classification using BBSLMs: the relative risk of marginal effects (RRME) and the odds ratio of marginal effects (ORME). The performance and interpretation of these new measures are illustrated in an application studying the risk of death within 48 hours of admission among individuals admitted to hospital with a myocardial infarction. The BBSLMs are shown to have better predictive ability than the logistic regression models, with the RRME:s and ORME:s of death for the main risk factor anterior infarct both being 1.8, comparable to the OR of 1.9 for the logistic regression model. The RRMEs and ORMEs are also shown to be more robust in terms of being applicable also for observations with missing values for some features.

## 1. Introduction

A common task in applied statistical analysis is to predict which of a number of distinct groups or categories an observation belongs to, i.e. a *classification* of the observation into a specific group or category. In a medical context, a binary classification, involving only two possible groups, is often of interest. Examples include predicting if a patient has cancer or not, if a disease requires surgery or not, or if a patient will survive a myocardial infarction or not. Traditionally, these classifications have been based on applying parametric statistical models, such as discriminant analysis or logistic regression, to the data set at hand, using a set of predictors (independent variables) to obtain a probability of the observation belonging to a particular group (outcome). An observation is then classified as belonging to a particular group (*success*) or not (*failure*) based on cut-off values applied to the probabilities obtained from the statistical model at hand.

However, during the last decades, a plethora of more and more sophisticated algorithm-based black box supervised learning models (BBSLMs) have been developed (see e.g. Hastie *et al.* [7] and Molnar [10]), often outperforming traditional parametric statistical models in terms of prediction accuracy [6,13]. Prominent examples include gradient boosting, random forests, and support vector machines. However, while BBSLMs often excel in terms of prediction accuracy, a common shortcoming is their lack of interpretability. In a medical setting, it is thus usually not only of interest to have a good accuracy in predicting if an individual will survive a myocardial infarction or not, but also to know how much higher the risk of death is for an individual having a particular risk factor of interest, after adjusting for age, sex, and important clinical characteristics. While traditional parametric statistical models such as logistic regression usually provides easy-to-interpret measures of such risks in the form of e.g. odds ratios (ORs), no similar easy-to-interpret effect measure has hitherto been available for BBSLMs. Breiman [2] calls this the *Occam dilemma*, i.e. the conflict between simplicity and accuracy: ‘Accuracy generally requires more complex prediction methods. Simple and interpretable functions do not make the most accurate predictors’. [2, p. 208]

### 1.1. Motivating application

A motivation for the present study was a study, discussed by Hilbe [8], focusing on the risk of death within 48 hours of admission among patients being admitted to hospital with a myocardial infarction. The primary aim of the study is to be able to accurately predict if a patient will die within 48 hours of admission or not, but as a secondary aim it is also of interest to estimate how much higher the risk of death within 48 hours of admission is for a patient depending on the presence of specific risk factors, in particular having an *anterior*-site infarction. While BBSLMs may be used to get excellent and accurate predictions of a patient's risk of death within 48 hours of admission, hitherto available methods have the shortcoming of not providing any effect measures estimating how much higher the risk of death within 48 hours of admission is depending on the presence of specific risk factors. The applied statistician has thus had to resort to instead using for example logistic regression models, which provide an easy-to-interpret effect measure for this setting in the form of ORs, but at the expense of giving less accurate predictions.

### 1.2. Aim and overview of the paper

To rectify this shortcoming in the application of BBSLMs in a classification setting like this, where no easy-to-interpret feature effect measures have hitherto been available, the present paper introduces the *relative risk of marginal effects* (RRME) and the *odds ratio of marginal effects* (ORME), two novel easy-to-interpret measures of how a change in one of the predictors or *features* in BBSLMs is effecting the outcome in a classification setting, after adjusting for the effect of the other features included in the model. Section 2 derives the formulas for the RRME and the ORME, while Section 3 illustrates the performance and interpretation of these new measure in an application to the above-mentioned data set of individuals admitted to hospital with a myocardial infarction. Additionally, the predictive ability of the BBSLMs and the magnitude of RRME and ORME are compared with the corresponding results from a logistic regression model. Section 4 concludes the paper with a discussion of the results and a suggestion of topics for future research.

## 2. Methodology

This section discusses the *partial dependence plot* (PDP) used in visualization of BBSLMs and shows how the RRME and ORME measures can be derived from and be used as feature effect measures for PDPs as well as BBSLMs in general.

### 2.1. Partial dependence plots

The partial dependence plot introduced by Friedman [4] is the most popular method for visualizing feature effects in BBSLMs [1]. Basically, a PDP provides a graphical depiction of the *marginal effect* a feature has on the predicted outcome of the model. In a classification setting, where the predicted outcome is given in the form of probabilities, the PDP shows the predicted probability of belonging to a particular group or category given different values of the feature of interest [10].

To formalize this, for a training data set, let *y* denote the scalar valued outcome of interest and x denote a *p* + 1 vector of predictor variables or features,

(1) $$x^{T}=\left(x_{0},x_{1},x_{2},\ldots ,x_{p}\right),$$

with $x_{p}=x\setminus x_{0}$ denoting the subvector containing the *p* variables $x_{i}$, i=1,2,…,p. Moreover, let $\hat{f}(x)$ denote the output of a BBSLM, i.e. $\hat{f}(x)$ is a general function of all features in the model which for the purpose of the present paper may be written as

(2) $$\hat{f}\left(x\right)=\hat{f}\left(x_{0},x_{p}\right).$$

Notably, $\hat{f}(x)$ is the fitted model that, in a classification setting, gives the predicted probability that *y* belongs to a particular group as a function of x [1]. The *partial dependence* of $\hat{f}(x)$ on $x_{0}$ may then be defined as

(3) $${\hat{f}}_{0}\left(x_{0}\right)=E_{x_{p}}\left[\hat{f}\left(x_{0},x_{p}\right)\right]=\int \hat{f}\left(x_{0},x_{p}\right)P_{p}\left(x_{p}\right)dx_{p},$$

where $P_{p}(x_{p})$ denotes the marginal probability density of $x_{p}$, i.e.

(4) $$P_{p}\left(x_{p}\right)=\int P\left(x\right)dx_{0},$$

where P(x) is the joint density over all features x. With $x_{\mathrm{jp}}$ denoting the values of $x_{p}$ for observation *j* in a training data set containing *n* observations, Equation (3) can be estimated by

(5) $${\bar{f}}_{0}\left(x_{0}\right)=\frac{1}{n}\sum_{j=1}^{n}\hat{f}\left(x_{0},x_{\mathrm{jp}}\right),$$

i.e. the effects of all the other features in the model are averaged out. A PDP thus shows the *marginal effect* of the feature $x_{0}$ on the predicted outcome by plotting the values of $x_{0}$ on the *x*-axis and ${\bar{f}}_{0}(x_{0})$ on the *y*-axis.

### 2.2. The relative risk of marginal effects (RRME)

To simplify the analyses, assume that we have a binary categorical feature $x_{0}\in \{0,1\}$, where 1 denotes the presence of a risk factor of interest and 0 denotes the absence of this risk factor. For the binary classification context of the present study, where the interest focuses on the probability that a patient dies within 48 hours of admission to hospital, we have a binary outcome y∈{0,1}, where 1 denotes that the patient dies within 48 hours of admission and 0 denotes that the patient is still alive 48 hours after admission. Using a Bernoulli loss function, we could then predict the *marginal effect* of $x_{0}$ on the probability that a patient dies within 48 hours of admission to hospital as

(6) $$P\left(y=1\;|\;x_{0}\right)={\bar{f}}_{0}\left(x_{0}\right).$$

In an epidemiological context, the *relative risk* is defined as the ratio between the probability of experiencing an event given the *presence* of a risk factor and the probability of experiencing an event given the *absence* of this risk factor. Utilizing this definition, we define the *relative risk of marginal effects* (RRME) as

(7) $$\mathrm{RRME}=\frac{P\left(y=1\;|\;x_{0}=1\right)}{P\left(y=1\;|\;x_{0}=0\right)}=\frac{{\bar{f}}_{0}\left(x_{0}=1\right)}{{\bar{f}}_{0}\left(x_{0}=0\right)}.$$

### 2.3. The odds ratio of marginal effects (ORME)

As is well known, the *odds* of experiencing an event is given by the ratio between the probability of experiencing the event and the probability of not experiencing the event. In the present context with a binary categorical feature $x_{0}\in \{0,1\}$ and an outcome y∈{0,1}, we thus have that

(8) $$\begin{array}{rl} \mathrm{odds}\left(y=1\;|\;x_{0}\right) & =\frac{P\left(y=1\;|\;x_{0}\right)}{1-P\left(y=1\;|\;x_{0}\right)}\\ & =\frac{{\bar{f}}_{0}\left(x_{0}\right)}{1-{\bar{f}}_{0}\left(x_{0}\right)}, \end{array}$$

which may be interpreted as the *odds of the marginal effect* of $x_{0}$. Equation (8) may then be used to calculate the *odds ratio of marginal effects* (ORME) of $x_{0}$, defined as

(9) $$\mathrm{ORME}=\frac{\mathrm{odds}\left(y=1\;|\;x_{0}=1\right)}{\mathrm{odds}\left(y=1\;|\;x_{0}=0\right)}=\frac{\frac{{\bar{f}}_{0}\left(x_{0}=1\right)}{1-{\bar{f}}_{0}\left(x_{0}=1\right)}}{\frac{{\bar{f}}_{0}\left(x_{0}=0\right)}{1-{\bar{f}}_{0}\left(x_{0}=0\right)}}.$$

### 2.4. Interpretation of RRME and ORME

Notably, RRME and ORME can be used as feature effect measures for PDPs, as well as BBSLMs in general, quantifying the strength of the association between the feature $x_{0}$ and the outcome *y*. Specifically, they give the strength of the marginal effect that the feature $x_{0}$ has on predicting that the outcome *y* belongs to the group of interest. They also give the direction of the association, with an RRME>1 or ORME>1 implying that the presence of the risk factor of interest (i.e. $x_{0}=1$) has a detrimental effect on the outcome in that it increases the risk of experiencing the event of interest (e.g. death within 48 hours of admission to hospital), while an RRME<1 or ORME<1 implies that the presence of the risk factor has a protective effect in that it decreases the risk of experiencing the event of interest.

While RRME and ORME as given in Equations (7) and (9) are only applicable to the case of a binary categorical feature $x_{0}$, they are easily extended to the case of a multinomial categorical feature $x_{0}\in \{0,1,2,3,\ldots \}$ by successively replacing $x_{0}=1$ in the numerators of (7) and (9) with $x_{0}=2$, $x_{0}=3$, and so on. Likewise, for the case of a continuous feature $x_{0}$, one could for example calculate the sample percentiles of $x_{0}$, replace $x_{0}=0$ in the denominators of (7) and (9) with $x_{0}=i$th percentile (which thus is used as the reference value), and then successively replace $x_{0}=1$ in the numerators of (7) and (9) with $x_{0}=j$th percentile, $x_{0}=k$th percentile, and so on. This would give the RRME and ORME, respectively, of the latter percentiles in relation to the *i*th percentile.

The odds ratio is arguably the most commonly used effect measure for the association between an exposure and a binomial outcome in epidemiologic research, most prominently due to its usefulness in the interpretation of logistic regression models. ORME thus has the advantage of being applicable and easily interpretable in the same context, and the results being comparable with those from logistic regression models. However, in a medical setting, the relative risk is usually the effect measure of primary interest, while the odds ratio is used as an approximation of the relative risk. The RRME thus has the advantage for BBSLMs of being a direct estimate of the relative risk. A drawback of the odds ratio is that it is sometimes misinterpreted as measuring the relative risk, and this misinterpretation may also affect the ORME.

## 3. Application

The study of the risk of death within 48 hours of admission among patients being admitted to hospital with a myocardial infarction described in the Introduction stems from a data set from the national Canadian cardiovascular registry which has been described and analyzed in detail by Hilbe [8, pp. 17, 25, 73, 113, 251]. It consists of 4696 patients admitted to hospital with a myocardial infarction, with the response variable of interest for the present study being *death,* which takes the value 1 if the patient died within 48 hours of admission to the hospital and 0 if the patient was still alive 48 hours after admission. The risk factor of main interest is the binary feature *anterior*, taking the value 1 if the patient had an anterior infarct and 0 if the patient had any other type of infarct. As secondary risk factors, we have the (pseudo-) continuous feature *age* (years), the binary feature *CABG*, where a value of 1 denotes that the patient has had a Coronary Artery Bypass Graft (CABG) surgery and 0 denotes that the patient has had a Percutaneous Transluminal Coronary Angioplasty (PTCA), and the four-level ordinal feature *Killip*. Killip is a measure of cardiovascular and cardiopulmonary severity, with each higher level representing an ascending degree of damage to the heart. Patients with Killip class I have normal functioning hearts. Notably, age was originally stored as a four-level categorical feature (≤60 years, 61–70 years, 71–80 years, >80 years). However, for pedagogical purposes, it was of interest to use age as a continuous feature. To this end, age was transformed to a (pseudo-) continuous feature ranging from 40 to 100 years using uniformly distributed random numbers for each age category, see Hilbe [8, p. 73f]. The actual exact ages of the patients were thus unknown.

### 3.1. Model estimation

In total, 2125 (45.3%) of the 4696 patients had an anterior infarct, of which 120 (5.6%) died within 48 hours of admission. Among the 2571 (54.7%) patients with any other type of infarct, 67 (2.6%) died within 48 hours of admission. Complete cases were available for all features except Killip class, for which 193 (4.1%) of the patients had missing values. To start with, 75% ( n=3521) of the 4696 patients were randomly selected to act as a training data set, with the remaining 1175 patients kept as a test data set. The random selection was performed using stratification on *death*, to ensure that an equal percentage (4.0%) of patients dying within 48 hours of admission was included in both the training and the test data set.

To illustrate the performance and interpretation of RRME and ORME, a stochastic gradient boosting (SGB) BBSLM [5] was estimated using the R package gbm version 2.1.9 [12] in R 4.4.1 (R Foundation for Statistical Computing, Vienna, Austria), applying a Bernoulli loss function fitted to 50,000 decision trees, with each tree having a depth of 1, a learning rate (shrinkage) of 0.001, a minimum number of 10 observations in the terminal nodes of the trees, and a subsampling rate (bag fraction) of 0.5. Class-stratified 10-fold cross-validation was used to estimate the optimal number of trees for the SGB model. Additionally, to compare the predictive ability of the SGB BBSLM and the magnitude of RRME and ORME with the corresponding results from a traditional parametric statistical model, a logistic regression model with *anterior*, *age* (years), *CABG*, and *Killip* class (using class I as reference category) as predictors and *death* as outcome was estimated for the training data set using the glm function in R 4.4.1 with a binomial error distribution and a logit link function. Finally, the SGB model with the optimal number of trees when applied to the training data set and the logistic regression model estimated from the training data set were both fitted to the test data set to obtain each patient's individual probability of dying within 48 hours of admission, as predicted from their values for *anterior*, *age*, *CABG*, and *Killip*.

The predictive abilities of the SGB and logistic regression models were evaluated using the *sensitivity* (in this context, the probability of being classified as dying within 48 hours of admission, given that one actually dies within 48 hours of admission) and *specificity* (in this context, the probability of being classified as not dying within 48 hours of admission, given that one does die within 48 hours of admission) measures in terms of the area under the receiver operating characteristics (ROC) curve (AUC). Since the ROC curve gives the balance between sensitivity and 1−specificity over all possible cut-off values, a higher AUC value implies an overall better predictive ability. Tests of differences between AUCs were performed using DeLong's test for paired ROC curves [3].

### 3.2. RRME and ORME calculations

**Figure 1.**
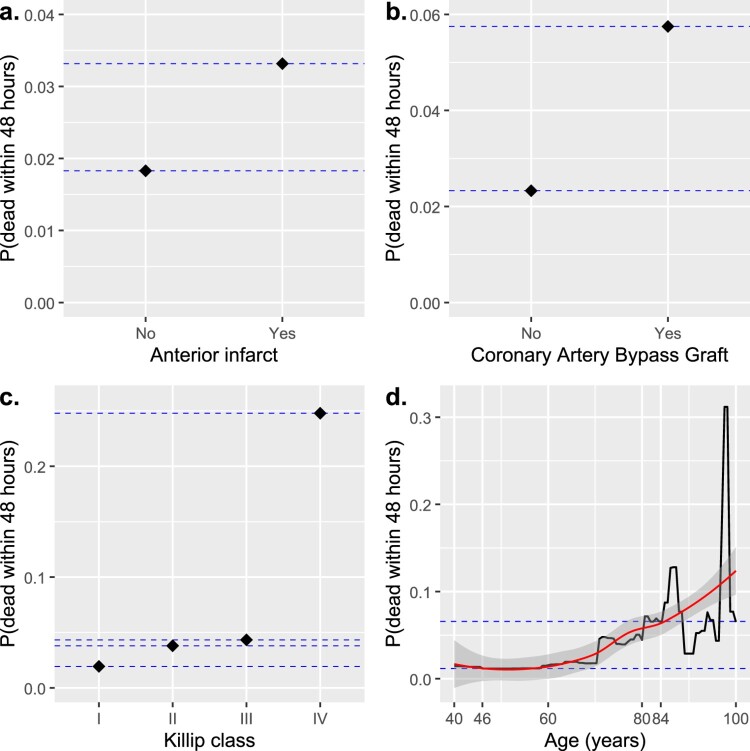
Partial dependence plots for the features (a) *anterior*, (b) *CABG* (Coronary Artery Bypass Graft), (c) *Killip*, and (d) *age* (including a local polynomial regression curve in red, with a 95% confidence band) for predicting the probability of death within 48 hours of admission for individuals admitted to hospital with a myocardial infarction included in the training data set with 3521 observations, based on the optimal stochastic gradient boosting model with 18,941 trees.

For the SGB model, the optimal number of trees was 18,941, with all four features having non-zero influence. Figure 1 gives the PDPs for predicting the probability of death within 48 hours for all features, while the exact probability values are given in Table 1. With this information, the RRMEs and ORMEs are easily calculated. For example, the RRME for *anterior* is given by

$$\begin{array}{rl} \mathrm{RRM}E_{\mathrm{anterior}} & =\frac{P\left(\mathrm{dead}\;\mathrm{within}\;48\;\mathrm{hours}\;|\;\mathrm{anterior}=\mathrm{Yes}\right)}{P\left(\mathrm{dead}\;\mathrm{within}\;48\;\mathrm{hours}\;|\;\mathrm{anterior}=\mathrm{No}\right)}\\ & =\frac{0.03316225}{0.01828856}=1.813 \end{array}$$

while the corresponding ORME for *anterior* is given by

$$\begin{array}{rl} \mathrm{ORM}E_{\mathrm{anterior}} & =\frac{\mathrm{odds}\left(\mathrm{dead}\;\mathrm{within}\;48\;\mathrm{hours}\;|\;\mathrm{anterior}=\mathrm{Yes}\right)}{\mathrm{odds}\left(\mathrm{dead}\;\mathrm{within}\;48\;\mathrm{hours}\;|\;\mathrm{anterior}=\mathrm{No}\right)}\\ & =\frac{\frac{P\left(\mathrm{dead}\;\mathrm{within}\;48\;\mathrm{hours}\;|\;\mathrm{anterior}=\mathrm{Yes}\right)}{1-P\left(\mathrm{dead}\;\mathrm{within}\;48\;\mathrm{hours}\;|\;\mathrm{anterior}=\mathrm{Yes}\right)}}{\frac{P\left(\mathrm{dead}\;\mathrm{within}\;48\;\mathrm{hours}\;|\;\mathrm{anterior}=\mathrm{No}\right)}{1-P\left(\mathrm{dead}\;\mathrm{within}\;48\;\mathrm{hours}\;|\;\mathrm{anterior}=\mathrm{No}\right)}}=\frac{\frac{0.03316225}{1-0.03316225}}{\frac{0.01828856}{1-0.01828856}}=1.841. \end{array}$$

The RRMEs and ORMEs for the other categorical features, *CABG* and *Killip*, are easily calculated in the same way. For the (pseudo-) continuous feature *age*, Figure 1d gives the curve for the local polynomial regression fit obtained using the R function loess. Notably, this curve approximately passes through the probabilities of death within 48 hours for patients aged 46 or 84 years, corresponding to the 10th and 90th percentiles, respectively, of the feature *age* in the training data set. Based on this, the RRME for *age* was calculated by first comparing the probabilities of death within 48 hours for patients aged 84 and 46 years, i.e.

$$\begin{array}{rl} \mathrm{RRM}E_{\mathrm{age},84-46} & =\frac{P\left(\mathrm{dead}\;\mathrm{within}\;48\;\mathrm{hours}\;|\;\mathrm{age}=84\right)}{P\left(\mathrm{dead}\;\mathrm{within}\;48\;\mathrm{hours}\;|\;\mathrm{age}=46\right)}\\ & =\frac{0.06577124}{0.01172691}=5.608574. \end{array}$$

In the second step, this value was transformed to measure the average feature effect per year as

(10) $$\mathrm{RRM}E_{\mathrm{age},\mathrm{years}}=\exp\left(\frac{\ln\left(\mathrm{RRM}E_{\mathrm{age},84-46}\right)}{84-46}\right)=1.046.$$

Likewise, the ORME for *age* was calculated by first comparing the odds of death within 48 hours for patients aged 84 and 46 years, i.e.

$$\begin{array}{rl} \mathrm{ORM}E_{\mathrm{age},84-46} & =\frac{\mathrm{odds}\left(\mathrm{dead}\;\mathrm{within}\;48\;\mathrm{hours}\;|\;\mathrm{age}=84\right)}{\mathrm{odds}\left(\mathrm{dead}\;\mathrm{within}\;48\;\mathrm{hours}\;|\;\mathrm{age}=46\right)}\\ & =\frac{\frac{P\left(\mathrm{dead}\;\mathrm{within}\;48\;\mathrm{hours}\;|\;\mathrm{age}=84\right)}{1-P\left(\mathrm{dead}\;\mathrm{within}\;48\;\mathrm{hours}\;|\;\mathrm{age}=84\right)}}{\frac{P\left(\mathrm{dead}\;\mathrm{within}\;48\;\mathrm{hours}\;|\;\mathrm{age}=46\right)}{1-P\left(\mathrm{dead}\;\mathrm{within}\;48\;\mathrm{hours}\;|\;\mathrm{age}=46\right)}}=\frac{\frac{0.06577124}{1-0.06577124}}{\frac{0.01172691}{1-0.01172691}}=5.933025, \end{array}$$

while in the second step, this value was transformed to measure the average feature effect per year as

(11) $$\mathrm{ORM}E_{\mathrm{age},\mathrm{years}}=\exp\left(\frac{\ln\left(\mathrm{ORM}E_{\mathrm{age},84-46}\right)}{84-46}\right)=1.048.$$

**Table 1.** Probability of death within 48 hours of admission for individuals admitted to hospital with a myocardial infarction according to the stochastic gradient boosting (SGB) model, together with relative risks of marginal effects (RRMEs), odds ratios of marginal effects (ORMEs), and odds ratios (ORs) for the SGB and logistic regression models, respectively, for predicting risk of death within 48 hours of admission.

	Stochastic gradient boosting			Logistic regression
Feature	P(dead withsin 48 hours)	RRME^a^	ORME^a^	OR^a^
Anterior infarct				
– Yes	0.03316225	1.813	1.841	1.941
– No	0.01828856	Reference	Reference	Reference
CABG				
– Yes	0.05748525	2.469	2.559	2.418
– No	0.02328079	Reference	Reference	Reference
Killip class				
– I	0.01938052	Reference	Reference	Reference
– II	0.03796595	1.959	1.997	1.912
– III	0.04333518	2.236	2.292	2.147
– IV	0.24784531	12.788	16.673	15.286
Age (years)		1.046^c^	1.048^c^	1.050
– 46^d^	0.01172691			
– 84^e^	0.06577124			

Note: CABG, Coronary Artery Bypass Graft. ${}^{a}$Results based on the optimal SGB model with 18,941 trees, using all *n* = 3521 observations in the training data set, of which 140 (4.0%) died within 48 hours of admission. ${}^{b}$Results based on *n* = 3374 (95.8%) of the 3521 observations in the training data set, of which 132 (3.9%) died within 48 hours of admission. ${}^{c}$Average value during ages 46 to 84 years. ${}^{d}$The 10th percentile for *age* in the training data set. ${}^{e}$The 90th percentile for *age* in the training data set.

### 3.3. Results

Table 1 gives the RRMEs, ORMEs, and ORs for the features in the SGB and logistic regression models, respectively, for predicting the risk of death within 48 hours of admission for individuals admitted to hospital with a myocardial infarction in the training data set. Notably, the RRME, ORME, and OR values are, in most cases, quite close to each other, for both categorical and continuous features. The RRME and ORME for *anterior*, the feature of main interest, are thus 1.813 and 1.841, respectively, compared to 1.941 for the OR, while the RRME and ORME for *age* are 1.046 and 1.048, respectively, compared to the OR value of 1.050. Accordingly, the conclusions about the effect of each feature on the risk of death within 48 hours of admission are practically the same. Holding the values of the other features constant, patients with an anterior infarct were thus 1.81 times as likely to die within 48 hours of admission compared to patients with any other type of infarct, according to the SGB model, while the odds of dying within 48 hours of admission were 1.84 and 1.94 times as large for patients with an anterior infarct compared to patients with any other type of infarct according to the SGB and logistic regression models, respectively. Likewise, for each additional year of age, a patient was 4.6% more likely to die within 48 hours of admission according to the SGB model, and had 4.8% and 5.0% higher odds of dying within 48 hours of admission according to the SGB and logistic regression models, respectively, holding the values of the other features constant. An exception is the value for *Killip* class IV, for which the RRME compared to the reference group with *Killip* class I is 12.788, a somewhat lower value than the corresponding values of 16.673 for the ORME and 15.286 for the OR. While a patient with Killip class IV was 12.8 times more likely to die within 48 hours of admission compared to a patient with Killip class I, the odds of dying within 48 hours of admission were thus 16.7 and 15.3 times larger for those with Killip class IV compared to those with Killip class I according to the SGB and logistic regression models, respectively, holding the values of all other features constant.

However, it should be noted that the results for the SGB and logistic regression models are not entirely comparable, since the SGB model easily incorporates missing data, and thus was estimated using all 3521 observations in the training data set, while the logistic regression model used only 3374 (95.8%) of the 3521 observations, since 147 patients had missing values for the *Killip* feature. The SGB model thus has the advantage over logistic regression of being more robust in that it can produce predictions for all patients, even for those with missing values for some features, the latter being an ubiquitous characteristic of real-world data.

Notably, the calculation of the average feature effect per year is affected by both the type of non-linear transformation applied and the percentiles used in the formulas. Using the 5th and 95th percentiles in Equations (10) and (11), corresponding to patients aged 43 and 93 years old, respectively, would thus give the RRME and ORME values 1.032 and 1.034, respectively. Likewise, using the 20th and 80th percentiles, corresponding to patients aged 51 and 77 years old, respectively, would result in RRME and ORME values of 1.051 and 1.052, respectively.

The Cramér's V measure of association between *anterior* and *CABG* was 0.025, between *anterior* and *Killip* 0.105, and between *CABG* and *Killip* 0.032, the point biserial correlation between *age* and *anterior* was 0.049 and between *age* and *CABG* 0.042, while Spearman's rank correlation between *age* and *Killip* was 0.134. Overall, the degree of feature overlap in the data set was thus low. Likewise, pairwise tests of interactions between the features in the logistic regression model showed significant interactions at the P-value<0.05 level only for interactions between *anterior* and *Killip* level III (*P* = 0.040) and between *age* and *Killip* level IV (*P* = 0.007), suggesting that the relationships between the included features and the outcome were largely captured by the model without interactions.

### 3.4. Predictions

**Figure 2.**
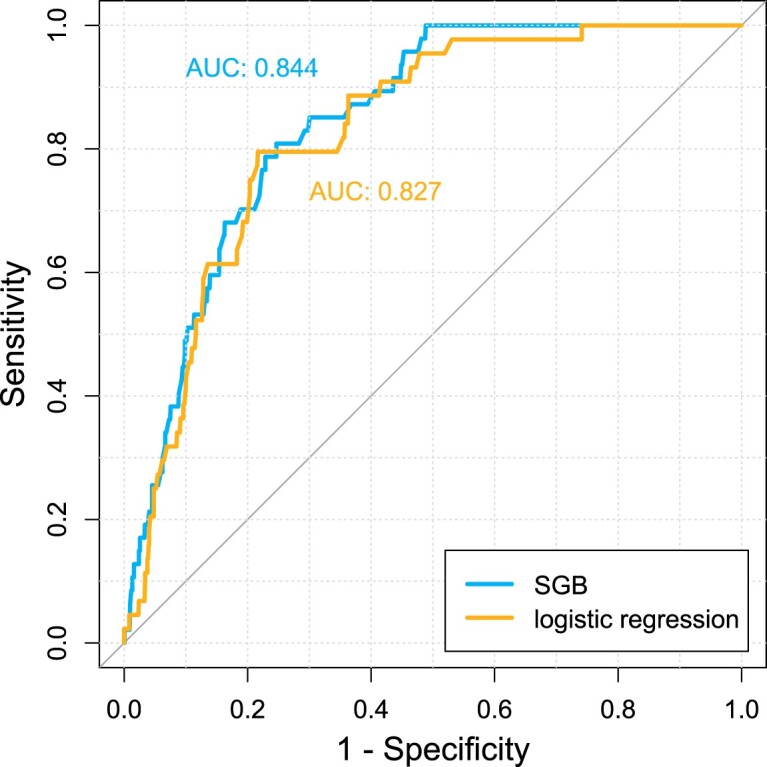
Receiver operating characteristics (ROC) curve for the optimal stochastic gradient boosting (SGB) model with 18,941 trees from the training data set applied to the 1175 patients in the test data set, together with the ROC curve from the corresponding logistic regression model applied to the 1129 (96.1%) patients with non-missing values in the test data set.

The ROC curve for the optimal SGB model from the training data set applied to the 1175 patients in the test data set is given in Figure 2, together with the ROC curve from the corresponding logistic regression model applied to the 1129 (96.1%) patients with non-missing values in the test data set. While the ROC curves follow each other quite closely, the curve for the SGB model is most of the time above the curve for the logistic regression model. This is also evident in the higher AUC value for the SGB model, at 0.844, compared to the AUC value of 0.827 for the logistic regression model. The SGB model is thus overall better at predicting the probability of death within 48 hours of admission to the hospital. Moreover, again it should be noted that the logistic regression model only works for patients without missing data on the *Killip* feature, while the SGB model works for all patients, regardless of the occurrence of missing data. It thus both have the advantage of being better at prediction and the advantage of being able to produce predictions for all patients.

Considering only the 1129 (96.1%) patients with non-missing values in the test data set, the SGB model reach an AUC value of 0.841, somewhat lower than the value of 0.844 for the full data set. While the DeLong test for paired ROC curves is not applicable for the full data set, where the SGB model utilizes all observations, it is applicable for this smaller data set with only non-missing values, where the observations utilized by the SGB and logistic regression models are paired. For the null hypothesis that there is no difference in AUC between the SGB and logistic regression models, we get a two-sided *P*-value of 0.170 from the DeLong test, meaning that we cannot reject the null hypothesis for this smaller data set with paired observations. However, it should be noted that while the full data set contains 47 cases (deaths), this smaller data set contains only 44 (93.6%) cases, thus giving less power for the hypothesis test, besides the disadvantage of having to discard patients with missing values for one variable. Overall, the advantages of using the SGB model over the logistic regression model for predictions are in this case obvious.

## 4. Discussion

The RRME and ORME feature effect measures for BBSLMs derived in the present paper should be useful additions to the field of interpretable machine learning in a classification setting, being applicable to both PDPs and BBSLMs in general. They should be particularly useful in a medical setting, where even clinicians with limited knowledge of statistical methodology often are acquainted with the concepts of relative risks and odds ratios, and thus intuitively will be able to interpret the meanings of RRMEs and ORMEs. Additionally, while PDPs have the advantage of visually showing the feature effects, since BBSLMs often involve hundreds of features, it will be hard to present and get an overview of PDPs for all these features. RRMEs and ORMEs, giving simple easy-to-interpret single numbers, should be more useful in this context. Moreover, PDPs are hard to use when the researcher is interested in comparing the feature effects from different studies, for which RRMEs and ORMEs should be more useful.

Compared to traditional parametric statistical models, a notable drawback of the RRME and ORME measures is the lack of easily applicable measures of uncertainty. While it in principle would be possible to use bootstrap methods to estimate the variance or confidence interval of RRMEs and ORMEs, this is in many cases not practically feasible due to the computationally demanding nature of BBSLMs and bootstrap methods, which may make the execution time necessary to obtain reliable estimates prohibitively long.

### 4.1. Related methods

While BBSLMs hitherto have lacked easy-to-interpret feature effect measures such as the RRME and ORME measures introduced in the present paper, there are a few measures available for measuring the importance or influence of the features included the model on the variation of $\hat{f}(x)$. One useful such measure is the relative influence of an individual feature on the variation of $\hat{f}(x)$ over the joint feature distribution, discussed by Friedman [4]. This measure is especially useful when the estimated relative influences are normalized to sum to 100, giving the normalized relative influence (NRI) values, which can be interpreted as giving the percentage of the variation in $\hat{f}(x)$ being explained by each individual feature [11]. RRMEs and ORMEs may thus be used together with NRIs to provide a fuller understanding of the predictions from BBSLMs.

The arguably most used method for BBSLMs is, however, the SHAP (SHapley Additive exPlanations) method introduced in the seminal paper by Lundberg and Lee [9]. SHAP is useful for visualizing the contribution of each feature to the probability of experiencing an outcome of interest for a particular observation. However, since the SHAP values are calculated separately for each observation, they do not give an interpretable feature effect measure for the overall model, in contrast to what is given by the RRME and ORME measures.

### 4.2. Topics for future research

While the present paper has focused on the basic case of binary classification, the methods developed herein should be easily extended to and applied in the context of multinomial classification. This is, however, a topic for future research. Likewise, since RRMEs and ORMEs are not applicable in the case of a continuous outcome, a further topic for future research is to develop similar methods for PDPs with a continuous outcome. Finally, developing practically feasible methods for measuring the variance of RRMEs and ORMEs would be a welcome contribution.

## Data Availability

The data set from the national Canadian cardiovascular registry with patients being admitted to hospital with a myocardial infarction used in this paper is available as the file HEARTR.DTA from https://www.stata.com/bookstore/LRM_DATA.ZIP.
